# Transcriptional Regulation of CYP2E1: Promoter Methylation in In Vitro Models and Human Liver Disease Samples

**DOI:** 10.3390/genes16080990

**Published:** 2025-08-21

**Authors:** Nina Komaniecka, Mateusz Kurzawski, Sylwia Szeląg-Pieniek, Joanna Łapczuk-Romańska, Mariola Post, Urszula Adamiak-Giera, Marek Droździk

**Affiliations:** 1 Department of Experimental and Clinical Pharmacology, Pomeranian Medical University, 70-111 Szczecin, Poland; nina.komaniecka@pum.edu.pl (N.K.); sylwia.szelag.pieniek@pum.edu.pl (S.S.-P.); joanna.lapczuk.romanska@pum.edu.pl (J.Ł.-R.); urszula.adamiak.giera@pum.edu.pl (M.D.); 2 Laboratory of Pharmacodynamics, Pomeranian Medical University in Szczecin, 71-899 Szczecin, Poland; 3 Department of General and Transplant Surgery, Pomeranian Regional Hospital, 71-455 Szczecin, Poland; 4 Department of Pharmacokinetics and Therapeutic Drug Monitoring, Pomeranian Medical University, 70-111 Szczecin, Poland; mariolapost@wp.pl

**Keywords:** cytochrome P450 2E1, epigenetic regulation, drug metabolism, liver pathology, human study

## Abstract

**Background/Objectives:** DNA methylation is a critical epigenetic mechanism involved in gene expression regulation. This study examines promoter methylation of *CYP2E1* in healthy liver, intestinal mucosa, as well as pathological liver samples, alongside in in vitro cell models. **Methods:** First, in tissue samples from the liver, duodenum, jejunum, and colon of healthy organ donors, *CYP2E1* promoter methylation was quantified using the EpiTect Methyl II PCR System, while gene expression was determined by quantitative real-time PCR. Then, in vitro experiments were performed using HepG2 and Caco-2 cell lines. Cells were treated with 5-Aza-2′-deoxycytidine to induce demethylation, with subsequent analysis of *CYP2E1* mRNA levels. Subsequently, promoter methylation was assessed via pyrosequencing, while gene expression was quantified using quantitative real-time PCR. **Results:** The analysis revealed statistically significant differences in the methylation patterns of the *CYP2E1* promoter between healthy liver and gastrointestinal tissues. In cell lines, treatment with 5-Aza-2′-deoxycytidine resulted in increased *CYP2E1* mRNA levels and demonstrated a strong negative correlation between promoter methylation and gene expression. However, in liver disease samples, differential methylation did not consistently translate into decreased *CYP2E1* expression. **Conclusions:** Although in vitro experiments support a regulatory role of promoter methylation in controlling *CYP2E1* expression, the clinical data indicate that additional factors may contribute to gene regulation in liver pathology.

## 1. Introduction

DNA methylation is a fundamental epigenetic mechanism that plays a pivotal role in the regulation of gene expression. Methylation predominantly takes place at genomic regions where cytosine is adjacent to the guanine nucleotide, commonly referred to as CpG dinucleotides [1]. As early as 1975, it was established that DNA methylation in promoter regions could interfere with DNA-binding proteins involved in transcription regulation, thereby influencing downstream gene expression. Additionally, DNA methylation was recognized as a key factor in the precise regulation of gene expression during embryogenesis. Since then, an increasing body of evidence has reinforced the crucial role of DNA methylation in embryonic development, gene regulation, and cellular memory [2,3]. DNA methylation is facilitated by a group of enzymes called methyltransferases, which are responsible for both de novo and maintenance methylation. Since methylation patterns are initially established during embryogenesis, this developmental stage is characterized by a high level of de novo methylation activity [1]. DNA methylation plays a crucial role in regulating gene expression influencing the accessibility of DNA to the gene expression machinery, both directly and indirectly. While it is often regarded as a repressive epigenetic mark, a substantial body of evidence indicates that it can also act as an activating mechanism, especially within the gene body [4].

DNA methylation in gene promoters interferes with transcription factor binding and inhibits the initiation of gene expression [1]. DNA is primarily methylated at CpG islands, and in the human genome, approximately 70% of gene promoters contain such islands [1,5]. However, it is important to recognize that this is not the only mechanism by which gene expression is regulated, as certain transcription factors can bind to DNA irrespective of its methylation status [1]. Furthermore, CpG methylation within the promoter, even outside of a transcription factor binding site, can also influence gene expression [6].

Although DNA methylation is the most extensively studied transcriptional/epigenetic modification, there are still gaps in our understanding of how it interacts with other epigenetic modifications and influences gene expression. Nonetheless, research on DNA methylation has shown that this epigenetic mark plays a crucial role in the processes underlying disease development. Mapping the DNA methylation patterns linked to disease states is essential for identifying and understanding the processes that drive disease development [1]. Systematic reviews indicate that both alcohol-associated and metabolic dysfunction-associated liver diseases share common DNA methylation changes affecting key pathways, including those related to cancer and PPAR signaling, highlighting the contribution of epigenetic regulation to liver pathology [7]. Epigenetic heterogeneity has been shown to emerge early in liver disease, altering the methylation of genes involved in hepatic functioning and carcinogenesis, which underlines the importance of epigenetic regulation in liver pathology [8]. Additionally, while DNA methylation can be stable and transferred to future cellular generations, it also remains reversible. The possibility of manipulating DNA methylation patterns has sparked interest in potential clinical applications [1]. This includes 5-Aza-2′-deoxycytidine (5-AZA), a cytidine analog that is incorporated into DNA. In contrast to cytidine, 5-azacytidine forms a permanent bond with DNA (cytosine-5)-methyltransferase 1 (DNMT1), leading to enzyme degradation and a general inhibition of DNA methylation [9].

The human enzyme CYP2E1, a member of the cytochrome P450 family, is responsible for the metabolism of over 80 toxic compounds. It is also instrumental in converting numerous pro-carcinogens and certain pharmaceuticals into highly reactive intermediates [10]. Additionally, this enzyme reduces oxygen into reactive oxygen species, which contribute to cellular damage, such as protein denaturation, enzyme inactivation, and DNA mutations [11]. The *CYP2E1* gene is upregulated by elevated ethanol levels. Its expression is subject to regulation through various mechanisms, including transcriptional and translational control, mRNA stabilization, and protein degradation [12].

It has long been established that *CYP2E1* expression is tissue-specific, and its enzymatic activity is particularly high in the liver [13]. Early investigations revealed that the methylation status of *CYP2E1* is closely associated with its expression, while subsequent studies documented significant interindividual variability in both its expression and functional activity [13,14]. In addition to methylation, other factors also influence the regulation of *CYP2E1* expression, including genetic polymorphisms [15], the nuclear receptor REV-ERBα ablation [16], or induction of cytochrome P450 due to oxidative stress (e.g., caused by ethanol exposure) [17]. The data suggest that *CYP2E1* expression and activity are influenced by various metabolic states, often leading to increased oxidative stress and cellular toxicity [17]. However, some conditions (like REV-ERBα ablation) may have protective effects [16]. The complex interplay between different regulatory mechanisms and metabolic states highlights the multifactorial nature of *CYP2E1* regulation in human liver cells [18]. Recent findings indicate that aging is accompanied by epigenetic alterations in *CYP2E1*, including increased DNA methylation and changes in histone acetylation in its regulatory regions, which correlate with reduced gene expression and altered drug metabolism in the liver [19]. The current state of knowledge remains insufficient regarding precise data on the regulation of *CYP2E1*, particularly in the context of liver pathology.

Given the enzyme’s pivotal role in disease progression and drug metabolism [20], our study focuses on characterizing whether liver pathologies are accompanied by distinct alterations in the *CYP2E1* methylation pattern. An altered promoter methylation pattern in pathological livers could potentially modulate drug metabolism—a critical aspect when treating such patients [20]. Research indicates that roughly 30% of patients with advanced liver failure experienced adverse drug reactions, and nearly 80% of these incidents might have been avoidable due to inappropriate dosing or the use of contraindicated medications [21]. These findings emphasize the clinical relevance of understanding methylation patterns in drug-metabolizing enzymes. Moreover, our own studies [22,23,24] have revealed significant differences in *CYP2E1* expression and protein abundance between healthy and diseased livers, and we confirmed its tissue specificity, noting high expression in the liver and much lower levels in the gastrointestinal tract [25]. Together, these observations have spurred our team on to further investigate potential variations in the *CYP2E1* promoter methylation pattern.

## 2. Materials and Methods

### 2.1. CYP2E1 DNA Methylation in Gastrointestinal Tract and Liver

Tissue samples were collected from the liver, duodenum, jejunum, and colon of 3 organ donors, as described previously [25]. DNA was extracted by means of a GeneMATRIX Tissue DNA Purification Kit (EURX, Gdańsk, Poland). DNA methylation of the *CYP2E1* promoter region was determined with an EpiTect Methyl II PCR System, including a primer assay for human *CYP2E1* (CpG island 102038, Qiagen, Germantown, MD, USA), according to the supplier’s protocol. The method is based on the detection of remaining input DNA after cleavage with a methylation-sensitive and/or a methylation-dependent restriction enzyme. Briefly, genomic DNA was aliquoted into four equal portions and subjected to mock (no enzyme), methylation-sensitive, methylation-dependent, and double restriction endonuclease digestion. After digestion, all samples were amplified with RT2 SYBR Green ROX qPCR Mastermix (Qiagen, USA) in separate 25 µL reactions, using a ViiA 7 Real-Time PCR System (Life Technologies, Carlsbad, CA, USA) with a 96-well block (43 cycles). Each sample was examined in duplicate. Finally, the relative amount of methylated and unmethylated DNA fractions was calculated based on the obtained ΔCT values, following the procedure recommended by the supplier, with the use of a Human Methylated and Non-methylated DNA Set (Zymo Research, Irvine, CA, USA) for data validation. The mRNA data for *CYP2E1* were available from a previous study [25].

### 2.2. CYP2E1 DNA Methylation in Caco-2 and HepG2 Cells

HepG2 and Caco-2 cell lines were cultured in DMEM (with 10% fetal bovine serum, additionally enriched with non-essential amino acids for Caco-2) in standard conditions (37 °C, 5% CO_2_). Cells for DNA analysis were cultured in 6-well plates (seeded at 50,000 per well), and for the gene expression analysis, in 24-well plates (10,000 per well). At 24 h after seeding, the medium was replaced with fresh DMEM containing 5-Aza-2′-deoxycytidine (5-AZA, Sigma-Aldrich, Darmstadt, Germany) at the following concentrations: 5 µM, 1.25 µM, 0.325 µM, and control medium without 5-AZA. For the subsequent days, the medium was replaced every day with a fresh one with the corresponding 5-AZA concentration. Isolation of DNA and RNA from the culture was carried out after a week of incubation. All the experiments were conducted in triplicate. Total RNA was extracted using the Direct-zol RNA MiniPrep Kit (Zymo Research, USA), while the GeneMATRIX Cell Culture DNA Purification Kit (Eurx, Poland) was used for DNA extraction. Following the isolation, DNA methylation of the *CYP2E1* promoter region was determined with the EpiTect Methyl II PCR System, as described for liver and gut samples, while *CYP2E1* gene expression (mRNA) was quantified by real-time PCR. Briefly, cDNA was synthesized using the SuperScript VILO cDNA Synthesis Kit (Thermo Fisher Scientific, Waltham, MA, USA), with 100 ng of total RNA for a reaction volume of 20 µL. Gene expression levels were determined in duplicate using TaqMan Fast Advanced Master Mix and a pre-validated TaqMan assay for human *CYP2E1* (manufacturer’s Assay ID: Hs00559367_m1) on the ViiA 7 Real-Time PCR System (Life Technologies, USA). The threshold value was set manually and mean CT values were recorded. Relative mRNA expression was calculated using the 2^−ΔΔCt^ method, normalized to the mean expression value obtained for the housekeeping genes *GAPDH* (Hs99999905_m1), *PPIA* (Hs04194521_s1), and *HPRT1* (Hs02800695_m1), and additionally to the mean value for the control (non-treated) cells.

### 2.3. CYP2E1 DNA Methylation in Liver Disease

Liver parenchymal tissue samples were also collected from patients subjected to liver transplantation and diagnosed with hepatitis C virus (HCV), primary biliary cholangitis (PBC), primary sclerosing cholangitis (PSC), alcoholic liver disease (ALD), Wilson’s disease (WD), and autoimmune hepatitis (AIH), as previously described [22,23,24]. Characteristics of all patient groups are given in Table 1. The study protocol was approved by the Bioethics Committee of the Pomeranian Medical University (approval number BN-001/11/07), and informed consent was obtained from all participants. All samples were collected between 2007 and 2018.

Total DNA was isolated from 25 mg of each tissue sample using the GeneMATRIX Tissue DNA Purification Kit (EURX, Poland). Bisulfite conversion of DNA was performed using a column method—EpiTect Fast DNA Bisulfite Kit (Qiagen, Venlo, The Netherlands). The conversion product constituted a template for the amplification reaction (PCR) of selected sequences containing CpG sites, using flanking pairs of primers, performed with use of a PCR kit (Qiagen, The Netherlands). The final step of the analysis was pyrosequencing the PCR products using a PyroMark Q48 Autoprep instrument (Qiagen, Netherlands), PyroMark Q48 Advanced CpG Reagents (Qiagen, The Netherlands), and CYP2E1 PyroMark CpG assay (Qiagen, The Netherlands, manufacturer’s assay ID: Hs_AL161645.3_01_PM), according to the manufacturer’s instructions. Each sample was analyzed in duplicate. A Human Methylated and Non-methylated DNA Set (Zymo Research, USA) was used as a positive and negative methylation control (100% and 0% of methylated CpG cytosines, respectively). Reaction design and primary analysis were performed using PyroMark Q48 Autoprep software (v.2.4.2). A quantitative result of the methylation of five cytosines for each sample (in CpG sequences) occurring within the CpG island in the *CYP2E1* gene promoter was expressed as a percentage of methylated cytosines.

The mRNA data for *CYP2E1* were available from previous studies [22,23,24]. However, in the present study, the number of cases was expanded to provide a more comprehensive analysis. Total RNA extraction and gene expression analysis was carried out using the same method as in our previous work [22]. For the available samples, CYP2E1 protein abundance was determined using the LC-MS/MS method, as previously described, with CYP2E1 protein abundance data originating from our earlier studies [22,23,24].

All procedures involving human subjects and/or human tissue samples at all stages of our research were conducted in accordance with the World Medical Association Declaration of Helsinki: Ethical Principles for Medical Research Involving Human Subjects and the International Committee of Medical Journal Editors (ICMJE) recommendations. The study protocol was reviewed and approved by the Bioethics Committee of the Pomeranian Medical University (approval number BN 001/11/07). Informed consent was obtained from all participants or their legal representatives prior to sample collection, and the privacy rights of participants were observed at all times. Human organs and tissues were procured in line with the WHO Guiding Principles on Human Cell, Tissue and Organ Transplantation and were not derived from executed prisoners or prisoners of conscience; autonomous consent free from coercion was obtained from all donors (or their next of kin—in case of organ donors).

The results were subjected to statistical analyses. The data distribution was determined using the Shapiro–Wilk test. The outliers were identified and removed following Tukey’s rule. Differences between study groups were evaluated using the nonparametric Kruskal–Wallis test for multiple comparisons with a post hoc Dunn’s test with Bonferroni correction or ANOVA with a post hoc Tukey’s HSD test accordingly. The homogeneity of variance was determined using Levene’s test. Correlations were established using the Spearman rank (rs) test. Statistical significance was set at *p* < 0.05. All statistical calculations were performed using the Statistica 13.3 Software Package (TIBCO Software Inc., Palo Alto, CA, USA).

## 3. Results

### 3.1. CYP2E1 DNA Methylation in Gastrointestinal Tract

Differences between percentages of methylated DNA in different sections of the gastrointestinal tract and liver were calculated by means of ANOVA with a post hoc Tukey’s HSD test (Table 2). The analysis demonstrated that the liver exhibits statistically significant differences from all segments of the gastrointestinal tract regarding both *CYP2E1* promoter DNA methylation and mRNA expression levels (*p* < 0.01). To evaluate this regulatory mechanism, in vitro experiments were conducted using HepG2 and Caco-2 cell lines.

### 3.2. CYP2E1 DNA Methylation in Caco-2 and HepG2 Cells

**Figure 1:**
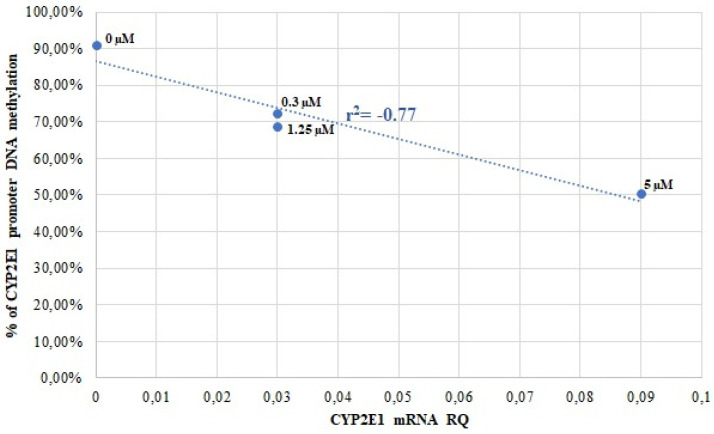
Correlation analysis of *CYP2E1* promoter DNA methylation with *CYP2E1* mRNA levels in HepG2 cells, treated with different 5-AZA concentrations (Spearman correlation coefficient r^2^ values).

**Figure 2:**
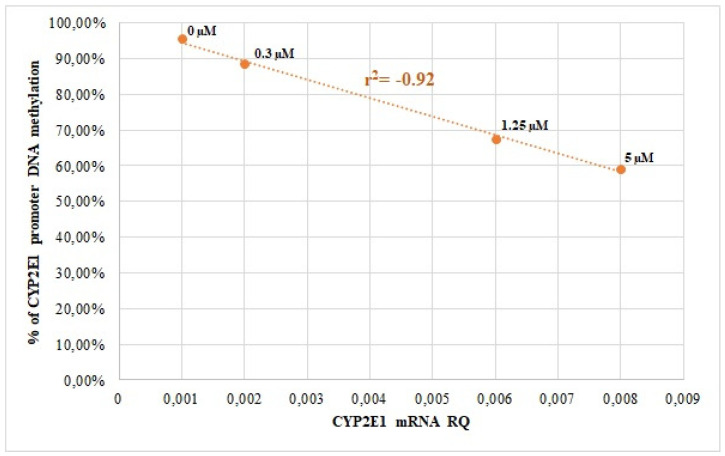
Correlation analysis of *CYP2E1* promoter DNA methylation with *CYP2E1* mRNA levels in Caco-2 cells, treated with different 5-AZA concentrations (Spearman correlation coefficient r^2^ values).

Our results show that *CYP2E1* expression in both HepG2 and Caco-2 cells was influenced by AZA-induced demethylation, with correlation coefficients of r^2^ = −0.77 (*p* < 0.01) for HepG2 and r^2^ = −0.92 (*p* < 0.0001) for Caco-2 (Figure 1 and Figure 2).

### 3.3. CYP2E1 DNA Methylation in Liver Disease

Despite previously observed significant differences in mRNA expression between healthy and pathological liver samples [22,23,24], our investigation did not reveal any statistically significant alterations in the methylation pattern within the diseased liver samples (Tables S1 and S2). Notably, the stage of liver disease also did not exhibit any significant association with the methylation pattern, as demonstrated in Table S2.

Nevertheless, we found a statistically significant negative correlation between the methylation level of the third CpG island in the *CYP2E1* promoter gene and *CYP2E1* mRNA level in all liver tissues (study group and control group altogether). However, this correlation is weak—r^2^ = −0.17. The data are shown in Table 3.

Despite earlier reports demonstrating differences in CYP2E1 protein abundance among various liver pathologies [22,23,24], statistical analysis revealed no significant correlation between *CYP2E1* promoter methylation and CYP2E1 protein abundance (Table S3).

## 4. Discussion

Similar findings confirming the tissue-specific expression of CYP2E1 are also available in the literature [13,26]; however, precise data regarding the relationship between *CYP2E1* promoter DNA methylation and mRNA expression, both in the gastrointestinal tract and the liver, are still lacking. Our findings substantiate the tissue-specific expression of CYP2E1 and support the hypothesis that its transcriptional regulation may be influenced by DNA methylation.

The results also confirm the well-known genome-wide demethylating effect of 5-AZA. Contrary to our results, Naselli et al. [15] demonstrated no consistent relationship between *CYP2E1* promoter DNA methylation and gene expression in vitro, although they employed different experimental approaches. The main differences include the use of Western blotting to assess *CYP2E1* expression, investigations in various cell lines—including HepG2 but not Caco-2—and the application of a different demethylating agent, ZCyd (another cytidine analogue). The results of Park et al. [27] support our findings, showing that *CYP2E1* DNA methylation is associated with reduced expression in human embryonic stem cell-derived hepatocyte (hESC-Hep) cells. However, their study focused on methylation within the gene body coding sequence rather than the promoter region. Additionally, in the latter study, DNA methylation was assessed using bisulfite sequencing and chromatin immunoprecipitation.

One available study shows *CYP2E1* methylation changes during healthy liver development, but specific methylation patterns in diseased liver tissue remain uncharacterized [28]. In that study, Vieira et al. documented that healthy liver fetal samples displayed a high methylation level in the 5′ flanking region, first exon, and first intron of *CYP2E1*. Methylation levels declined in neonatal livers, a change that accompanies increases in gene expression. In contrast, other studies of the diseased liver samples such as hepatocellular carcinoma (HCC), non-alcoholic fatty liver disease (NAFLD), and alcoholic liver disease (ALD) report broad epigenetic alterations but do not provide details on *CYP2E1*-specific methylation changes [29,30,31]. Gao et al. [29] identified four patterns of aberrant methylation in HCC, suggesting that methylation changes occur progressively from adjacent tissue to HCC. Murphy et al. [31] found a predominance of hypomethylation in advanced NAFLD, with specific patterns associated with disease progression, while Zeybel et al. [30] further supported disease-specific methylation patterns in NAFLD and ALD.

Botto et al. [13] observed a relationship between hypomethylation and hypoexpression of *CYP2E1* in healthy liver samples, but the statistical significance of these findings was not mentioned in that study. Moreover, the Southern blot method used by those authors is not sufficiently precise or sensitive compared to the methods employed in our research. Furthermore, Bonder et al. [26] analyzed 181 adult and 14 fetal healthy liver samples using bisulfite conversion and methylation arrays and identified 3238 significant methylation–expression associations (*p* < 0.05), with 58.4% of these correlations being negative. Contrary to our research, neither of the above-mentioned studies compared healthy with diseased liver tissue, so the evidence does not address differences associated with liver disease. Despite these reports, the results of our research appear to contradict the notion that the *CYP2E1* promoter methylation pattern is associated with decreased *CYP2E1* expression in liver diseases.

Study limitations include the relatively small number of healthy organ donors available for gastrointestinal tract and liver comparisons, which may restrict statistical power and generalizability. While functional relevance is suggested, the absence of enzyme activity measurements in pathological liver samples prevents confirmation of the biological impact of the observed methylation patterns. Furthermore, this study focuses solely on promoter methylation, leaving unresolved the potential contributions of other regulatory mechanisms, such as histone modifications, noncoding RNAs, or chromatin accessibility.

## 5. Conclusions

Overall, our findings confirm tissue-specific *CYP2E1* expression associated with the gene promoter methylation level; however, they indicate that *CYP2E1* DNA methylation is not the determinant of downregulated *CYP2E1* mRNA expression and CYP2E1 protein abundance in liver pathology. Despite a significant correlation between the level of DNA methylation in the promoter region of the *CYP2E1* gene and its expression in vitro, such a correlation has not been confirmed in vivo. This indicates a complex, multifactorial regulation of *CYP2E1* expression and CYP2E1 protein abundance in liver tissue, extending beyond a simple relationship with DNA methylation. Future studies could focus on identifying alternative regulatory mechanisms controlling *CYP2E1* expression in liver pathology, such as transcription factor networks, chromatin accessibility, noncoding RNAs, or post-translational modifications. Investigating how these factors interact with metabolic and pathological states could provide deeper insights into *CYP2E1* regulation and its role in drug metabolism under disease conditions. Additionally, integrating multi-omics approaches may uncover novel biomarkers or therapeutic targets for liver dysfunction.

## Figures and Tables

**Table 1:** Patient characteristics.

	ALD *n* = 21	HCV *n* = 58	PBC *n* = 12	PSC *n* = 7	WD *n* = 8	AIH *n* = 17	CTRL *n* = 29
Sex (M/F) *	11M, 10F	30M, 28F	1M, 11F	6M, 1F	5M, 3F	7M, 10F	17M, 11F
Age (mean ± SD)	51 ± 7	56 ± 8	58 ± 7	42 ± 12	36 ± 12	47 ± 17	60 ± 14
Child–Pugh scale	A1, B8, C12	A29, B21, C8	A4, B3, C5	A5, B2, C0	A1, B2, C5	A5, B5, C7	N/A
Total bilirubin [mg/dL] (mean ± SD)	4.5 ± 4.2	1.7 ± 1.2	5.1 ± 5.8	4.3 ± 5.6	12.0 ± 14.2	3.6 ± 3.8	0.6 ± 0.3
Albumin [g/dL] (mean ± SD)	3.0 ± 0.6	3.4 ± 0.6	3.3 ± 0.6	3.7 ± 0.5	3.2 ± 1.3	3.4 ± 0.4	3.6 ± 0.7
PT [s] (mean ± SD)	16.1 ± 2.4	14.0 ± 4.0	13.3 ± 1.9	12.3 ± 3.0	35.9 ± 15.8	14.4 ± 5.3	13.0 ± 3.2
INR (mean ± SD)	1.5 ± 0.2	1.3 ± 0.3	1.2 ± 0.2	1.2 ± 0.3	2.9 ± 1.9	1.4 ± 0.6	1.2 ± 0.2

* M—male, F—female. Abbreviations: ALD—alcoholic liver disease patients, HCV—hepatitis C patients, PBC—primary biliary cholangitis patients, PSC—primary sclerosing cholangitis patients, WD—Wilson’s disease patients, AIH—autoimmune hepatitis patients, CTRL—control patients, N/A—not applicable.

**Table 2:** Percentage of methylated DNA within the analyzed promoter region of CYP2E1 gene and CYP2E1 mRNA expression in different sections of the digestive tract and liver (mean ± SD).

*CYP2E1*	Methylated DNA (%)	mRNA (Relative Quantity)
liver	24.50 ± 7.89	58.27 ± 19.50
duodenum	65.17 ± 7.71 *	0.02 ± 0.02 *
jejunum	74.30 ± 5.33 *	0.01 ± 0.01 *
colon	78.23 ± 1.53 *	0.00 ± 0.00 *

* *p* < 0.01; statistical significance in relation to liver group, from ANOVA with post hoc Tukey’s HSD test.

**Table 3:** Correlation analysis of CpG cytosine methylation with *CYP2E1* mRNA levels in the liver tissue (Spearman correlation coefficient r^2^ values).

Methylated Cytosine	ALD	HCV	PBC	PSC	WD	AIH	ALL	CTRL	ALL + CTRL
C1	0.09	−0.03	0.03	−0.46	0.71	−0.09	−0.01	−0.24	−0.07
C2	−0.24	−0.02	0.01	−0.57	0.00	−0.25	−0.09	−0.26	−0.13
C3	−0.02	−0.11	0.08	−0.57	0.37	−0.18	−0.10	−0.36	−0.17 *
C4	−0.04	−0.02	−0.11	−0.57	−0.14	−0.22	−0.04	−0.15	−0.12
C5	−0.01	−0.01	0.07	−0.57	−0.03	−0.35	−0.06	−0.19	−0.14

* *p* < 0.05. Abbreviations: ALD—alcoholic liver disease patients, HCV—hepatitis C patients, PBC—primary biliary cholangitis patients, PSC—primary sclerosing cholangitis patients, WD—Wilson’s disease patients, AIH—autoimmune hepatitis patients, ALL—all patients from the study group, CTRL—control group patients, ALL + CTRL—all patients from study and control groups.

## Data Availability

The original contributions presented in this study are included in the article/Supplementary Materials. Further inquiries can be directed to the corresponding author.

## Supplementary Materials

The following supporting information can be downloaded at https://www.mdpi.com/article/10.3390/genes16080990/s1, Table S1: Percentage of the consecutive methylated CpG cytosines within the analyzed CpG is-land in the promoter region of *CYP2E1* gene in liver pathology and control group, Table S2: Percentage of the consecutive methylated CpG cytosines within the analysed CPG island in the promoter region of *CYP2E1* gene in liver pathology according to the Child-Pugh scale and control group, Table S3: Correlation analysis of CpG cytosine methylation with CYP2E1 protein levels in the liver tissue (Spearman correlation coefficient r2 values).

## Abbreviations

The following abbreviations are used in this manuscript:

HCV	Hepatitis C Virus
PBC	Primary Biliary Cholangitis
PSC	Primary Sclerosing Cholangitis
ALD	Alcoholic Liver Disease
WD	Wilson’s Disease
AIH	Autoimmune Hepatitis
HCC	Hepatocellular Carcinoma
NAFLD	Non-Alcoholic Fatty Liver Disease
