# Supplementary material for: Transcriptional Regulation of CYP2E1: Promoter Methylation in In Vitro Models and Human Liver Disease Samples
Source: Genes (Basel). 2025 Aug 21;16(8):990. doi: 10.3390/genes16080990 (PMC12385311; doi:10.3390/genes16080990)
Supplement: Supplementary file 1 [file genes-16-00990-s001.zip › genes-3824743-supplementary.pdf]

Table S1. Percentage of the consecutive methylated CpG cytosines within the analyzed CpG island in the promoter region of *CYP2E1* gene in liver pathology and control group.

Methylated cytosine	ALD	HCV	PBC	PSC	WD	AIH	ALL	CTRL
**C1**	8.2±15.4%	7.5±11.6%	5.7±13.7%	6.5±10.2%	3.2±0.5%	5.6±10.8%	6.2±12.4%	7.6±5.5%
**C2**	18.1±12.3%	16.0±15.1%	10.7±17.1%	11.9±12.4%	10.7±14.1%	12.5±13.5%	13.3±16.4%	14.3±9.6%
**C3**	14.5±10.2%	12.0±10.9%	9.3±14.6%	10.0±9.5%	8.4±2.2%	9.3±14.2%	10.8±12.7%	10.6±7.1%
**C4**	23.4±12.9%	22.0±16.0%	16.1±19.5%	20.1±10.8%	14.6±4.5%	14.8±18.2%	20.0±17.7%	20.3±8.7%
**C5**	15.9±9.9%	15.5±11.4%	9.3±13.2%	12.6±9.5%	9.6±2.6%	10.7±12.8%	14.0±13.1%	12.7±7.5%

Differences vs. control group calculated by means of ANOVA Kruskal-Wallis (% of methylated cytosine, median ± IQR). Abbreviations: ALD – alcoholic liver disease patients, HCV – hepatitis C patients, PBC – primary biliary cholangitis patients, PSC – primary sclerosing cholangitis patients, WD – Wilson's disease patients, AIH – autoimmune hepatitis patients, ALL – all patients from the study group, CTRL – control group patients. No significant differences were observed.

Table S2. Percentage of the consecutive methylated CpG cytosines within the analysed CpG island in the promoter region of *CYP2E1* gene in liver pathology according to the Child-Pugh scale and control group

Methylated cytosine	A	B	C	CTRL
**C1**	9.2±10.1%	6.0±11.8%	5.0±14.5%	7.6±5.5%
**C2**	15.7±14.5%	11.9±16.5%	11.4±19.5%	14.3±9.6%
**C3**	13.1±10.2%	10.3±11.8%	9.4±17.3%	10.6±7.1%
**C4**	20.9±14.6%	20.0±18.4%	17.8±20.2%	20.3±8.7%
**C5**	15.2±9.7%	13.9±13.7%	11.5±16.1%	12.7±7.5%

Abbreviations: A – patients with Child-Pugh A scale, B – patients with Child-Pugh B scale, C – patients with Child-Pugh C scale, CTRL – control group patients. No significant differences were observed.

Table S3: Correlation analysis of CpG cytosine methylation with CYP2E1 protein levels in the liver tissue (Spearman correlation coefficient r2 values).

**Methylated cytosine**	**ALD** *n=19*	**HCV** *n=57*	**PBC** *n=6*	**PSC** *n=5*	**WD** *n=4*	**AIH** *n=14*	**ALL** *n=105*	**CTRL** *n=20*	**ALL+CTRL** *n=125*
C1	0.15	-0.01	-0.20	0.60	-0.20	0.08	0.05	-0.10	-0.02
C2	-0.11	0.00	-0.09	0.50	-0.80	-0.00	0.03	-0.10	-0.03
C3	0.05	0.02	-0.20	0.50	-0.40	0.18	0.06	-0.13	-0.03
C4	0.06	0.04	-0.09	0.50	-1,00	0.07	0.07	0.06	-0.01
C5	-0.02	-0.03	-0.66	0.50	-0.80	-0.07	0.01	-0.04	-0.04

Abbreviations: ALD – alcoholic liver disease patients, HCV – hepatitis C patients, PBC – primary biliary cholangitis patients, PSC – primary sclerosing cholangitis patients, WD – Wilson's disease patients, AIH – autoimmune hepatitis patients, ALL – all patients from the study group, CTRL – control group patients, ALL+CTRL – all patients from study and control group.
